# Early‐life adverse events and risk of inflammatory bowel disease: A Scandinavian birth cohort study

**DOI:** 10.1002/jpn3.70537

**Published:** 2026-08-21

**Authors:** Ida Sigvardsson, Ketil Størdal, Henrik Imberg, Elisabeth Fernell, Christopher Gillberg, Johnny Ludvigsson, Karl Mårild

**Affiliations:** ^1^ Department of Pediatrics Institute of Clinical Sciences, Sahlgrenska Academy Gothenburg Sweden; ^2^ Department of Pediatric Research, Faculty of Medicine University of Oslo Oslo Norway; ^3^ Children's Center Oslo University Hospital Oslo Norway; ^4^ Statistiska Konsultgruppen Sweden AB Gothenburg Sweden; ^5^ Department of Molecular and Clinical Medicine, Institute of Medicine, Sahlgrenska Academy University of Gothenburg Gothenburg Sweden; ^6^ Gillberg Neuropsychiatry Centre, Institute of Neuroscience and Physiology University of Gothenburg Gothenburg Sweden; ^7^ Crown Princess Victoria Children's Hospital Region Östergötland Linköping Sweden; ^8^ Division of Pediatrics, Department of Biomedical and Clinical Sciences Linköping University Linköping Sweden; ^9^ Department of Pediatrics Queen Silvia Children's Hospital Gothenburg Sweden

**Keywords:** ABIS, childhood adversity, life events, MoBa, stressful events

## Abstract

**Objectives:**

The experience of childhood stressful or adverse life events is believed to imprint on immunological pathways and, therefore, might have lasting effects on inflammatory bowel disease (IBD) development. This study aimed to examine the association between experiences of early‐life adverse events and later IBD risk.

**Methods:**

We followed children in the Norwegian Mother, Father, and Child Cohort Study (MoBa) and All Babies in Southeast Sweden cohort (ABIS) from birth (1997–2009) and linked cohort data to national patient registers to identify IBD diagnoses through the end of 2024 and 2023, respectively. We included 111,293 participants with data on adverse life events reported during pregnancy, by age 1, or by age 3. Adverse life events included adverse maternal or familial events, such as divorce or loss of a family member, based on questionnaire data from the two prospective birth cohorts. Cox regression was used to estimate adjusted hazard ratios (aHRs) for IBD in children exposed versus unexposed to adverse maternal or family events, accounting for parental and child characteristics. Cohort‐specific estimates were pooled using a random effects model.

**Results:**

We identified 697 IBD events over 2,255,642 person‐years of follow‐up. Experience of maternal adverse events in pregnancy, reported by 22% of the mothers, was not associated with offspring IBD; pooled aHR 1.00 (95% confidence interval [CI] = 0.57–1.76), compared with no experience. Neither experience of adverse family events by age 1 year (pooled aHR 0.96, 95% CI = 0.80–1.15), nor by age 3 years (pooled aHR 0.99, 95% CI = 0.83–1.17) was associated with later IBD.

**Conclusions:**

This bi‐national prospective cohort study found no support for the hypothesis that experiences of early‐life adverse events increase the risk of IBD.

## Abstract (highlights)

**What is Known:**

- Psychological stress‐related factors are known to impact the disease course of established inflammatory bowel disease (IBD).
- Childhood stressful or adverse life events are believed to imprint on immunological pathways and could therefore affect future IBD development.

**What is New:**

- Using prospectively collected data from two large‐scale birth cohorts, we examined the association between experiences of early‐life adverse events, specifically those occurring during the prenatal period and the first 3 years of life, and later IBD development.
- This study found no support for the experience of early‐life adverse events as a major risk factor for IBD development.

## Graphical abstract

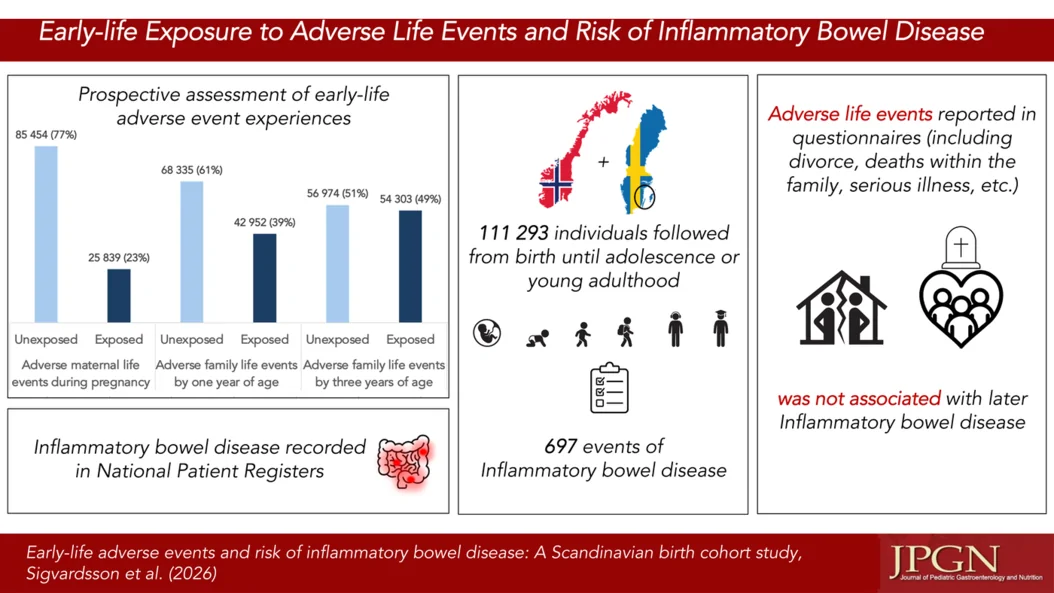

## 1 INTRODUCTION

Early‐life stressful or adverse events, like parental divorce, loss of a family member, or severe illness, may have lasting effects on the hypothalamic‐pituitary‐adrenal (HPA) axis,^1^ gut microbiota,^2^ and immune dysregulation, including proinflammatory cytokine production.^3^ In the All Babies In Southeast Sweden (ABIS) cohort, children who experienced family stressors had elevated hair cortisol levels, contributing to immune response imbalances.^4^ Early‐life adverse events have been linked to the subsequent development of several immune‐mediated diseases, such as type 1 diabetes (T1D) and other autoimmune disorders.^5^ Psychological stress has consistently been shown to exacerbate inflammatory bowel disease (IBD) symptoms and negatively influence disease course, like increasing relapse risk,^6^, ^7^, ^8^, ^9^ acting through pathways in the central nervous system^10^ and potentially the enteric nervous system, demonstrated in murine models.^11^ Nevertheless, it remains uncertain if psychological stress may also have an etiological role in IBD development.

Evidence linking early‐life adverse events to IBD development is limited to retrospective or cross‐sectional studies, which are liable to recall bias and reverse causation (Supporting Information S1: Table S1). It may be challenging to determine whether stress is a causative factor or a consequence of IBD using retrospective data. We aimed to use prospectively collected data from two Nordic birth cohorts to assess the role of early‐life adverse events and later risk of IBD, and its subtypes, Crohn's disease (CD) and ulcerative colitis (UC).

## 2 METHODS

### 2.1 Ethics statement

Parents provided written informed consent for their children upon enrollment in each cohort. ABIS was approved by the research ethics committees at Linköping University [Li 287‐96] and Lund University [Lu 83‐97]. The establishment of MoBa and initial data collection were based on a license from the Norwegian Data Protection Agency and approval from the Regional Committees for Medical and Health Research Ethics (REK), and this substudy received additional approval (no. 153328).

### 2.2 Study population

This study is based on two prospective birth cohorts: The Norwegian, Mother, Father and Child Cohort Study (MoBa) and the ABIS cohort. MoBa invited pregnant women from 50 out of 52 hospitals with delivery units across Norway. About 114,000 children born between 1999 and 2009 were recruited to MoBa (participation rate 41%). ABIS invited parents of children born between October 1, 1997, and October 1, 1999, across southeastern Sweden. During this period, 17,055 children were recruited to ABIS (participation rate 79%). The two birth cohorts followed children through childhood using repeated questionnaires, primarily administered to mothers, on the children's development, health, diet, social factors, and parental health‐related factors. This study included children with a valid personal identity number (PIN),^12^ provided to all long‐term residents in Norway and Sweden, enabling linkage of the cohort data to national health registers. For participation, we also required data on at least one of the exposures described below.

### 2.3 Outcomes

We identified IBD from diagnostic records in the Swedish and Norwegian National Patient Registers (NPRs). We defined IBD as ≥2 International Classification of Diseases 10th Revision (ICD‐10) codes for the disease (Supporting Information S1: Table S2). This definition has shown a high positive predictive value (PPV) of ≥93% in Sweden and Norway,^13^, ^14^, ^15^ and an estimated negative predictive value of 100% for childhood‐onset IBD.^15^ Subtype‐specific codes defined CD and UC. Individuals with a combination of ICD‐10 codes for CD, UC, and IBD‐Unclassified (IBD‐U) within the last 2 years were classified as having IBD‐U, that is, colonic IBD that could not readily be distinguished between CD and UC.

### 2.4 Exposures

This study examined the term “adverse” (i.e., stressful) life experiences at three time points:

1. Adverse maternal events during pregnancy.
2. Adverse family events by age 1 year (defined by either maternal, child, and/or family experiences).
3. Adverse family events by age 3 years (defined by either maternal, child, and/or family experiences).

In ABIS, adverse maternal events during pregnancy were retrieved from the at‐birth questionnaire, through binary response options of Yes/No for the experience of difficult life events, with examples given such as the death of a relative, and divorce. Data on early‐life adverse family events were retrieved from questionnaires administered at 12 months and 2.5–3 years after birth, reflecting serious or dramatic events experienced by the child or the family (Yes/No/Don't know), exemplified by death, divorce, or a new caregiver. In the 12‐month questionnaire and in a diary kept during the child's first year of life, parents also reported adverse life events in free text.

In MoBa, data on adverse life events during pregnancy were obtained from the 30‐week pregnancy questionnaire, and data on early‐life adverse events were obtained from the 6‐, 18‐, and 36‐month questionnaires. At all three time points, specific response options were available for events including divorce/separation, conflicts with the family/friends, serious illness/injuries of oneself or someone close, involvement in a serious accident/fire/robbery, and the loss of someone close. Experience of adverse maternal or family events included any of these. All questionnaires in MoBa included a question about the perceived difficulty of the events. We primarily considered “Painful/difficult” or “Very painful/difficult” as adverse life events in MoBa; in secondary analyses, we used a more stringent definition of adverse events, limiting adverse events to only those perceived as “Very painful/difficult” in MoBa to harmonize exposure across cohorts.

We harmonized data across cohorts for events during pregnancy, by 1 year, and by 3 years. Due to the granularity of questionnaire data available, our exposures were dichotomized (Yes [exposed]/No [unexposed]). Supporting Information S1: Table S3 provides a complete description of the questionnaire data and harmonization between the cohorts.

### 2.5 Covariates

Based on previous literature we identified characteristics believed to be associated with early‐life adverse events and/or IBD: the child's sex, parental IBD and origin (Norwegian/Swedish or other), maternal age at delivery, educational level (9–11, 12, or ≥13 years),^16^ smoking during pregnancy,^17^ and immune‐mediated comorbidities (T1D/insulin‐treated diabetes, autoimmune thyroid disease, or rheumatoid arthritis; Supporting Information S1: Table S4 and Supporting Information S1: Figure S1).

### 2.6 Other data

Psychosocial factors may influence both the exposure to early‐life adverse events and their impact on the child. We therefore included information on maternal social support at birth and at the child's age 3 years (high/low) and feelings of sadness/depression after birth (yes/no) (Table 1 and Supporting Information S1: Table S3).

**Table 1:** Participant characteristics in the MoBa and ABIS cohorts according to the experience of, and data availability on, adverse maternal or family events at any time point.

	MoBa			ABIS		
Characteristics	All with data at any time point *N* = 95,220	No adverse life event across all time points *N* = 22,072	Adverse life event experience at any time point *N* = 46,861	All with data at any time point *N* = 16,073	No adverse life event across all time points *N* = 4365	Adverse life event experience at any time point *N* = 4430
Sex						
Male	48,717 (51%)	11,061 (50%)	24,194 (52%)	8334 (52%)	2213 (51%)	2311 (52%)
Female	46,503 (49%)	11,011 (50%)	22,667 (48%)	7739 (48%)	2152 (49%)	2119 (48%)
Age (year) at the end of follow‐up^a^						
Mean (SD)	19.4 (2.2)	19.3 (2.1)	19.5 (2.2)	25.2 (1.3)	25.2 (1.1)	25.21 (1.3)
Median (IQR)	19.3	19.2	19.3	25.3	25.3	25.3
	(17.7, 21.1)	(17.7, 20.8)	(17.8, 21.2)	(24.8, 25.8)	(24.8, 25.8)	(24.8, 25.8)
Calendar year of birth						
1997	–	–	–	1655 (10%)	438 (10%)	471 (11%)
1998	–	–	–	8528 (53%)	2258 (52%)	2400 (54%)
1999	30 (<0.1%)	0 (0%)	11 (<0.1%)	5890 (37%)	1669 (38%)	1559 (35%)
2000	1747 (2%)	1 (<0.1%)	811 (2%)	–	–	–
2001	3680 (4%)	427 (2%)	1769 (4%)	–	–	–
2002	7803 (8%)	1980 (9%)	3933 (8%)	–	–	–
2003	11,408 (12%)	2654 (12%)	5706 (12%)	–	–	–
2004	12,252 (13%)	3005 (14%)	6298 (13%)	–	–	–
2005	13,951 (15%)	3425 (16%)	6898 (15%)	–	–	–
2006	15,495 (16%)	3707 (17%)	7540 (16%)	–	–	–
2007	14,188 (15%)	3286 (15%)	6894 (15%)	–	–	–
2008	11,763 (12%)	2824 (13%)	5672 (12%)	–	–	–
2009	2903 (3%)	763 (4%)	1329 (3%)	–	–	–
IBD diagnosis only including cases diagnosed with IBD after 1 year						
IBD	546 (0.6%)	124 (0.6%)	289 (0.6%)	151 (0.9%)	42 (1%)	42 (0.9%)
CD	227 (0.2%)	51 (0.2%)	127 (0.3%)	56 (0.3%)	17 (0.4%)	10 (0.2%)
UC	185 (0.2%)	44 (0.2%)	95 (0.2%)	85 (0.5%)	24 (0.5%)	27 (0.6%)
Maternal feelings of depression in the child's first year of life						
No	74,454 (89%)	20,698 (95%)	36,115 (85%)	7367 (85%)	3733 (86%)	2688 (83%)
Yes	8873 (11%)	1080 (5%)	6399 (15%)	1327 (15%)	599 (14%)	559 (17%)
Missing	11,893	294	4347	7379	33	1183
Maternal social support at birth						
Low	2857 (3%)	498 (2%)	1464 (3%)	122 (0.8%)	20 (0.5%)	42 (1%)
High	88,939 (97%)	21,400 (98%)	43,490 (97%)	15,555 (99%)	4311 (100%)	4269 (99%)
Missing	3424	174	1907	396	34	119
Maternal social support at 3 years of age						
Low	1691 (3%)	590 (3%)	969 (3%)	472 (5.5%)	180 (4%)	244 (8%)
High	52,999 (97%)	21,064 (97%)	28,524 (97%)	8182 (95%)	4148 (96%)	2986 (92%)
Missing	40,530	418	17,368	7419	37	1200
Any breastfeeding duration						
0–<4 months	11,492 (13%)	2175 (10%)	6537 (15%)	1466 (13%)	436 (10%)	553 (16%)
4–<6 months	7640 (9%)	1596 (7%)	3995 (9%)	1110 (10%)	378 (9%)	375 (11%)
≥6 months	67,096 (78%)	18,301 (83%)	33,119 (76%)	8387 (77%)	3351 (80%)	2614 (74%)
Missing	8992	0	3210	5110	200	888
Maternal age^b^						
<25 years	10,118 (11%)	1477 (7%)	5728 (12%)	2517 (16%)	469 (11%)	822 (19%)
25–34 years	68,420 (72%)	16,447 (75%)	33,084 (71%)	11,338 (72%)	3254 (76%)	3021 (69%)
35–44 years	16,633 (17%)	4136 (19%)	8023 (17%)	1960 (12%)	567 (13%)	514 (12%)
Missing	49	12	26	258	75	73
Maternal education level						
9–11 years	7039 (7%)	849 (4%)	4018 (9%)	1352 (9%)	223 (5%)	433 (10%)
12 years	27,820 (29%)	5213 (24%)	14,575 (31%)	8794 (55%)	2389 (55%)	2396 (55%)
>13 years	59,953 (63%)	15,923 (72%)	28,085 (60%)	5659 (36%)	1742 (40%)	1522 (35%)
Missing	408	87	183	268	11	79
Parental IBD^c^						
No IBD	93,004 (98%)	21,564 (98%)	45,751 (98%)	15,879 (99%)	4310 (99%)	4367 (99%)
IBD	2216 (2%)	508 (2%)	1110 (2%)	194 (1%)	55 (1.3%)	63 (1%)
Parental origin^d^						
Norwegian/Swedish	81,985 (90%)	19,872 (91%)	40,179 (89%)	14,111 (89%)	3992 (92%)	3886 (89%)
Other country	9323 (10%)	1847 (9%)	4828 (11%)	1694 (11%)	360 (8%)	461 (11%)
Missing	3912	353	1854	268	13	83
Maternal immune‐mediated comorbidities						
No	91,402 (96%)	21,318 (97%)	44,682 (95%)	15,507 (96%)	4222 (97%)	4253 (96%)
Yes	3818 (4%)	754 (3%)	2179 (4.6%)	566 (4%)	143 (3%)	177 (4%)

*Note*: See Supporting Information S1: Table S4 for definitions of covariates and data sources. Baseline characteristics were summarized using means with SD or medians with IQR for continuous variables, and counts with percentages for categorical variables. Percentages ≥1 were rounded to the closest integer.

Abbreviations: ABIS, All Babies in Southeast Sweden; CD, Crohn's disease; IBD, Inflammatory bowel disease; IQR, interquartile range; MoBa, The Norwegian Mother, Father, and Child Cohort Study; SD, standard deviation; UC, ulcerative colitis.

^a^ Equaled the age at study end (end of 2024 in MoBa; end of 2023 in ABIS) or age at diagnosis for IBD, CD, and UC cases.

^b^ Grouped in analyses to six age groups: 15–19, 20–24, 25–29, 30–34, 35–39, 40–44, combined for table presentation.

^c^ At least one parent with an IBD diagnosis.

^d^ Based on parental native language in MoBa and country of birth in ABIS, and considered “other country” if at least one parent reported another native language than Norwegian, or not being born in Sweden, respectively.

### 2.7 Statistical analysis

Follow‐up started at the time of exposure assessment (i.e., at birth, at age 1 year, or at 3 years, depending on the exposure) and ended at the time of IBD diagnosis or censoring at the end of data capture (December 31, 2023, in ABIS and December 31, 2024, in MoBa). Associations between exposures and IBD outcomes were analyzed using Cox proportional hazards regression models fitted separately within each cohort. To account for non‐independence between siblings, cluster‐robust standard errors were applied. Cohort‐specific hazard ratios were pooled using random‐effects meta‐analysis according to the DerSimonian–Laird method.^18^ Heterogeneity between the cohorts was assessed using Cochran's *Q*‐test and *I* ^2^ statistics^19^ and was low or moderate for most exposures.

In the main analyses, missing exposure and covariate data were handled using multiple imputation by chained equations under the assumption of missing at random. Exposure data (provided that at least one exposure measurement was available) and covariate data were imputed using 20 imputed datasets. Available case analyses were additionally performed to test the robustness of the findings from the multiple imputation analyses. All statistical analyses were conducted using R software (R Foundation for Statistical Computing, Vienna, Austria), versions 4.2.2 and 4.5.0, packages meta, metafor, mice, and survival.^20^, ^21^, ^22^, ^23^, ^24^, ^25^

In pre‐planned sensitivity analyses, we additionally adjusted for breastfeeding duration,^26^ diet quality at 1 year of age,^27^ and antibiotic use by 1 year,^28^ environmental factors that may influence early‐life adverse events and have, albeit inconsistently, been associated with IBD risk. We also conducted sensitivity analyses for the risk for childhood‐onset IBD, defined as IBD diagnosed before 18 years of age.

## 3 RESULTS

### 3.1 Study population

A total of 111,293 participants across the MoBa and ABIS cohorts were included with data on early‐life adverse events (Supporting Information S1: Figure S2). Participants with and without data on early‐life adverse events were comparable, except for a slightly higher maternal educational level among participating children (Supporting Information S1: Table S5). The mean age of participants at the end of follow‐up was 19.4 (SD 2.2) years in MoBa and 25.2 (SD 1.3) years in ABIS (Supporting Information S1: Table S6). Over 2,255,642 person‐years of follow‐up, 697 participants developed IBD (MoBa 546 [0.57%]; ABIS 151 [0.94%]). The incidence rate (IR) of IBD was 29.5 (confidence interval [CI] = 27.1–32.1) per 100,000 person‐years in MoBa, and the age‐ and sex‐standardized IR was 28.1 (95% CI = 19.3–40.9) per 100,000 person‐years in ABIS (Supporting Information S1: Table S7).

### 3.2 Adverse life events during pregnancy

Almost one in five mothers reported adverse life events during pregnancy (25,010/111,293 [22%]; Supporting Information S1: Table S6). Estimations differed markedly between the cohorts (*I* ^2^ > 65%; Cochran's *Q* test *p* < 0.10). In ABIS, about 7% of the mothers reported adverse life events during pregnancy. Adverse life events during pregnancy were not associated with IBD (adjusted hazard ratios [aHR] 0.68 [95% CI = 0.36–1.30]), and there were no statistically significant results in the ABIS cohort across all analyses (Figure 1 presents estimates for IBD; Figure 2 for CD; Figure 3 for UC). In MoBa, 25% of the mothers reported adverse life events during pregnancy. In MoBa, experience of adverse life events during pregnancy was statistically significantly associated with IBD in the child (aHRs 1.24; 95% CI = 1.03–1.50), particularly CD (aHR 1.59; 95% CI = 1.20–2.09), but not UC (aHR 1.02; 95% CI = 0.73–1.42). In pooled analysis, maternal adverse life events during pregnancy were not associated with IBD risk in the child (aHR 1.00; 95% CI = 0.57–1.76; Figure 1). Subtype‐specific pooled analyses yielded similar estimates: aHR 0.91 (95% CI = 0.23–3.64) for CD and aHR 0.93 (95% CI = 0.62–1.38) for UC (Figures 2 and 3). In secondary analyses using a more stringent definition of adverse life events in MoBa, 9% of mothers reported experiencing adverse life events during pregnancy. Using the more stringent definition of adverse life events in MoBa yielded non‐statistically significant associations across all analyses (Supporting Information S1: Table S8), with similar estimates across the cohorts (*I* ^2^ < 30%; Cochran's *Q* test, *p* > 0.10).

**Figure 1:**
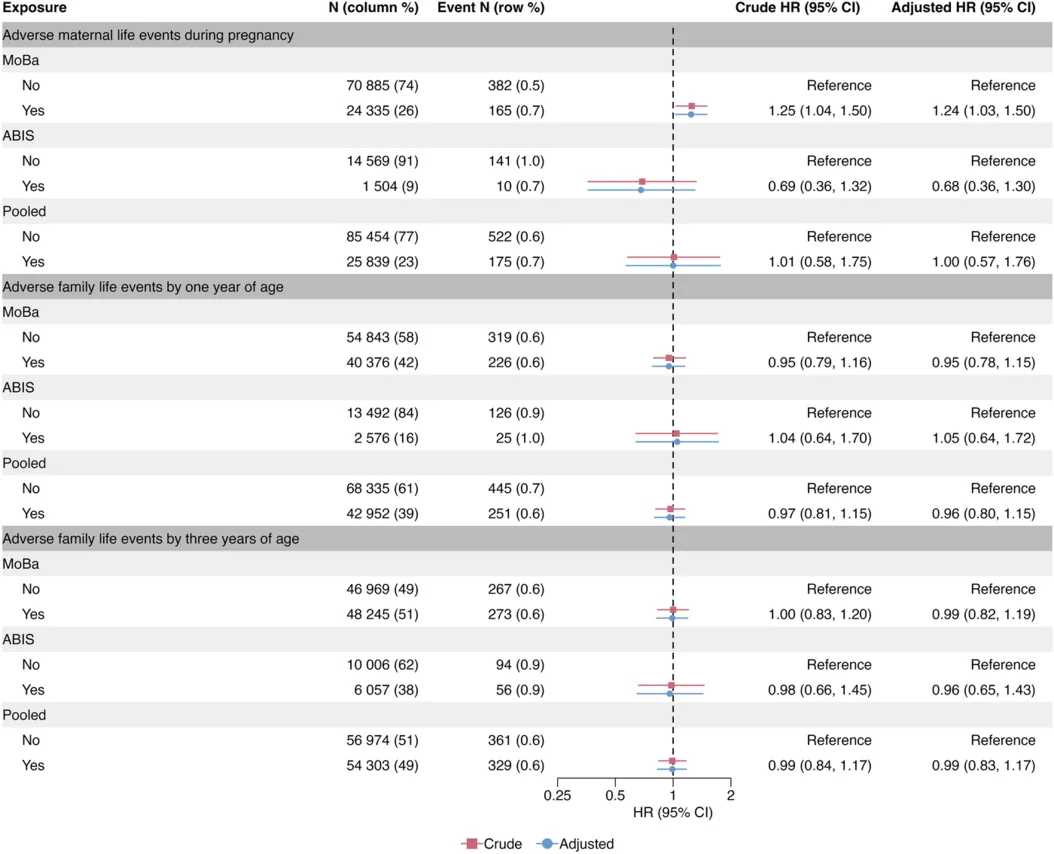
Associations between early‐life adverse events and IBD in the ABIS and MoBa cohorts and pooled across cohorts. Points and horizontal lines represent HRs with 95% CIs. Numbers represent mean values based on 20 imputations. Adjusted model included child's sex, parental IBD and origin, maternal age at delivery, educational level, smoking during pregnancy, and comorbidities. ABIS, All Babies in Southeast Sweden; CIs, confidence intervals; HRs, hazard ratios; IBD, inflammatory bowel disease; MoBa, The Norwegian Mother, Father, and Child Cohort Study.

**Figure 2:**
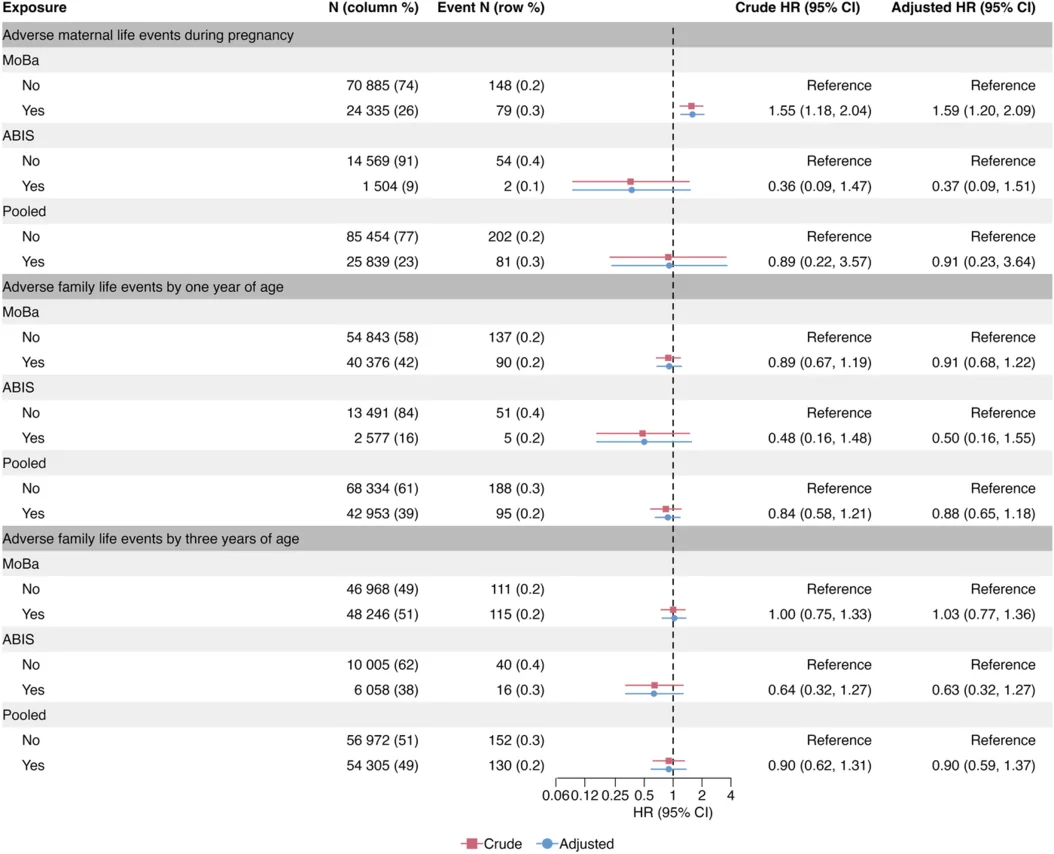
Associations between early‐life adverse events and Crohn's disease in the ABIS and MoBa cohorts, pooled across cohorts. Points and horizontal lines represent HRs with 95% CIs. Numbers represent mean values based on 20 imputations. Adjusted model included child's sex, parental IBD and origin, maternal age at delivery, educational level, smoking during pregnancy, and comorbidities. ABIS, All Babies in Southeast Sweden; CIs, confidence intervals; HRs, hazard ratios; IBD, inflammatory bowel disease; MoBa, The Norwegian Mother, Father, and Child Cohort Study.

**Figure 3:**
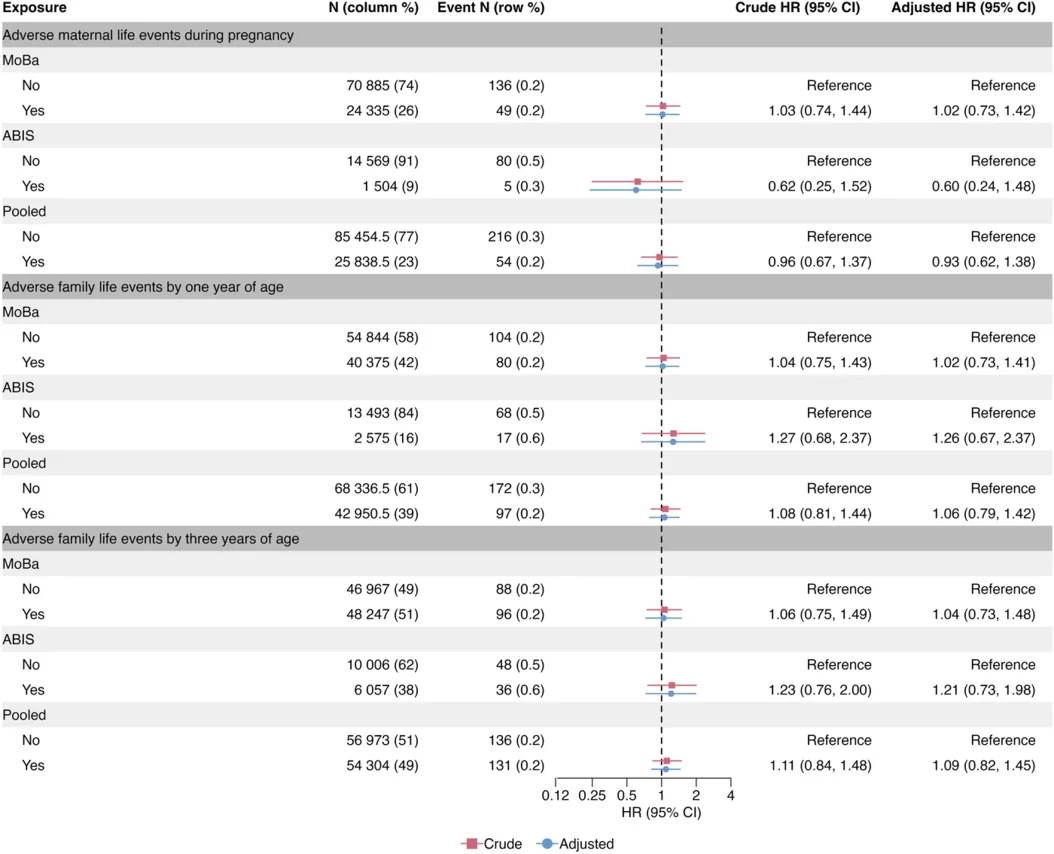
Associations between early‐life adverse events and ulcerative colitis in the ABIS and MoBa cohorts, pooled across cohorts. Points and horizontal lines represent HRs with 95% CIs. Numbers represent mean values based on 20 imputations. Adjusted model included child's sex, parental IBD and origin, maternal age at delivery, educational level, smoking during pregnancy, and comorbidities. ABIS, All Babies in Southeast Sweden; CIs, confidence intervals; HRs, hazard ratios; IBD, inflammatory bowel disease; MoBa, The Norwegian Mother, Father, and Child Cohort Study.

### 3.3 Adverse family life events in the first 3 years of life

The great majority of mothers did not report sadness or depression within the child's first year of life (89%), and most experienced high social support at birth (97%) and at 3 years of age (97%; Table 1). However, mothers with higher frequencies of feelings of depression, low education, and low age reported more often adverse family life events (Table 1). Across cohorts, approximately one‐third of mothers reported adverse family life events by the child's age of 1 year (29%; Supporting Information S1: Table S6). Adverse family life events by age 1 year were reported by 16% in ABIS and 42% in MoBa (secondary analyses using a more stringent definition of adverse life events resulted in 18% in MoBa; Supporting Information S1: Table S8). Cohort‐specific estimates for adverse family life events by age 1 and by age 3 were consistently not associated with later IBD (Figures 1, 2, 3). In pooled analyses, adverse family life events by age one were not associated with IBD (aHR 0.96; 95% CI = 0.80–1.15), CD (aHR 0.88; 95% CI = 0.65–1.18), or UC (aHR 1.06; 95% CI = 0.79–1.42; Figures 1, 2, 3) in the child. Similarly, pooled analyses of adverse family events by age 3 years (36% frequency, Supporting Information S1: Table S6) yielded aHRs of 0.99 (95% CI = 0.83–1.17) for IBD, 0.90 (95% CI = 0.59–1.37) for CD, and 1.09 (95% CI = 0.82–1.45) for UC (Figures 1, 2, 3). Pooled secondary analyses based on the stricter definition of adverse life events in MoBa were all statistically nonsignificant (Supporting Information S1: Table S8).

### 3.4 Available case analyses

Available case analyses included 108,218 individuals for adverse maternal life events during pregnancy, 84,985 by age 1 year, and 71,039 by age 3 years (Supporting Information S1: Figure S3). Similar to primary analyses on imputed data, available‐case analyses of adverse maternal life events during pregnancy in MoBa yielded increased aHRs for IBD (1.25; 95% CI = 1.03–1.51) and CD (1.56; 95% CI = 1.17–2.09), but no significant associations were observed in ABIS (data not shown). Pooled risk estimates for IBD were close to one: adverse maternal events during pregnancy, aHR 1.02 (95% CI = 0.60–1.74); adverse events by age 1 year, aHR 0.97 (95% CI = 0.81–1.17); adverse family events by age 3 years, aHR 0.94 (95% CI = 0.78–1.14). Similar null findings were found for pooled CD and UC analyses (Supporting Information S1: Table S9).

### 3.5 Sensitivity analyses

Additional adjustments for breastfeeding duration, diet quality at 1 year, and antibiotic use by 1 year yielded similar estimates as the primary analyses, with pooled HRs close to one for IBD, CD, and UC (Supporting Information S1: Table S10). Restricting the follow‐up to childhood‐onset IBD resulted in unchanged estimates (e.g., maternal adverse life events during pregnancy and IBD, aHR 0.99 (95% CI = 0.39–2.52); Supporting Information S1: Table S11).

## 4 DISCUSSION

This study is the first to use prospectively collected data from two population‐based cohorts to assess the association between early‐life adverse events and the development of IBD. We found hazard ratios for IBD and its subtypes consistently close to one across pooled analyses and sensitivity analyses. Overall, the findings do not support that early‐life adverse events are a major risk factor for the development of IBD.

The role of stress in established IBD, primarily on clinical flares, has been widely examined. However, few studies have investigated early‐life stressors and their impact on IBD development.^29^, ^30^, ^31^, ^32^, ^33^, ^34^ Most,^29^, ^32^, ^33^, ^34^ but not all,^30^, ^31^ of these retrospective case‐control studies have reported associations between childhood stressors and later IBD development. Most examined stressful events in older children, whereas our present study examined events in the first 3 years of life, including pregnancy. Gnat et al. reported no associations between “traumatic events” before the age of 17 years, such as the death of a close friend or family member, or major parental upheaval, and IBD risk. However, reported severity of total trauma was lower in healthy controls compared to IBD patients (odds ratio [OR] 0.89; 95% CI 0.81–0.97).^30^ In a Danish population‐based study, Jakobsen et al. found parental divorce more common in patients with childhood‐onset IBD diagnosed before 15 years of age, compared to controls (adjusted OR 1.7; 95% CI 1.0–2.9).^32^ In a case‐control study of 98 IBD cases, Bednarikova and colleagues found childhood life stressors, family violence, and physical abuse two to five times more prevalent among patients with IBD, compared to controls.^29^ Similarly, Strisciuglio et al. found “stressing events” within the family more often reported in pediatric patients with CD (46%) and UC (55%) than controls (35%); univariable OR 1.6 (95% CI 1.0–2.6) for CD, and 2.2 (95% CI 1.4–3.5) for UC. However, associations were not confirmed in multivariable analysis.^33^ Inconsistencies across earlier studies may partly reflect differences in the timing of exposure assessment and exposure definitions, such as parental divorce,^31^, ^32^ violence,^29^ or overall adverse events.^33^

Adverse events in childhood have been examined in relation to other immune‐mediated diseases like T1D^5^, ^35^, ^36^ and asthma,^37^, ^38^ conditions sharing epidemiological traits with IBD. In previous ABIS studies, parental exposure to “serious life events” or parental‐reported child experiences at 5–6 and 8–9 years of age were associated with increased risk of T1D.^35^ Life events, especially stressful for the child, were associated with the development of diabetes‐related autoantibodies,^39^ although events in the child's first year of life were not linked to the development of early‐onset T1D.^36^ Another Swedish cohort study found an association between parental experience of “serious life events” in the child's first 2 years of life and T1D, but not for events during pregnancy.^40^ In MoBa, maternal “negative life events” during pregnancy and 6 months after birth were associated with asthma development in children at age seven.^37^

The prevalence of adverse events differed between the MoBa and ABIS cohorts (e.g., 42% vs*.* 15% by 1 year of age). One possible explanation for these differences concerns the questions used to capture adverse events: perceived severity of an adverse event may vary depending on whether it is directed at the mother, the child, or the whole family. Secondary analyses, restricting the severity of adverse events in MoBa to those perceived as very painful or difficult, resulted in more similar frequencies of adverse life events across the cohorts, suggesting differences in questionnaire formulation or interpretation. It is also possible that sociocultural variations in the interpretation and reporting of adverse events may have influenced the frequency of adverse events. It is also essential to acknowledge the high subjectivity of adverse experiences and their perceived severity. The amount of social support from relatives and friends also affects perceived stress,^41^ including that arising from an adverse event. Most parents in our study reported high social support during their child's early life.

This study has several strengths. One is the prospective design, with exposure data collected in close temporal proximity to its occurrence and before disease development; this improves the precision of exposure assessments and minimizes recall bias and the risk of reverse causation. Although residual confounding cannot be ruled out in observational studies, another strength was the ability to comprehensively adjust for a wide range of relevant confounders, such as parental IBD and socioeconomic factors, and report data on maternal feelings of depression and social support. These are factors closely related to adverse life events or IBD, but that have not always been accounted for in previous works. Using two Scandinavian cohorts comprising more than 111,000 individuals followed throughout childhood provided substantial follow‐up time. Both the Norwegian and Swedish NPRs are of high quality,^42^, ^43^ with PPVs for our IBD definition as high as 94% in Norway^14^ and 93% in Sweden.^13^, ^15^

We also acknowledge several limitations. Although we assessed the overall experience of any adverse events, we were unable to examine the association with cumulative exposure to adverse events, which has been associated with other diseases.^44^ Second, limited granularity of the questionnaire data prevented us from distinguishing whether adverse events primarily affected the mother, the child, or the family, or from assessing the nature and severity of individual events (including specific details on how a divorce was carried out). This may have resulted in non‐differential exposure misclassification, potentially biasing estimates toward the null. Similarly, we could not define the degree of closeness to a deceased relative (e.g., losing a parent or grandparent), which could influence the child differently. Third, we lacked information on several potentially important early‐life determinants of adverse events, such as socioeconomic instability, parental alcohol or substance misuse, and mental health disorders, which may represent sources of unmeasured confounding. Mothers in MoBa were older compared to mothers in the general population of Norway, but this has not been shown to materially influence exposure‐outcome associations.^45^

This study did not evaluate the connection between adverse life events and neurodevelopmental disorders in relation to IBD development. It could be an essential aspect of childhood stress, as adversity in childhood and neurodevelopmental disorders often coexist, putting the child at double risk of poor health outcomes later in life.^46^ Associations between IBD and neurodevelopmental disorders, such as Attention Deficit Hyperactivity Disorder and Autism Spectrum Disorder, have been explored with diverse results.^47^, ^48^

## 5 CONCLUSION

While the impact of adverse family events likely varies considerably between individuals, the consistent null findings in this prospective binational birth cohort suggest that early‐life adverse events are not a significant population‐level risk factor for the development of IBD in the young. However, more modest associations overall or with specific adverse events not captured in this study cannot be ruled out.

## CONFLICT OF INTEREST STATEMENT

Karl Mårild received payment for lectures for Pfizer and is a sub‐investigator at a clinical trial financed by Pfizer. Elisabeth Fernell was supported by a research stipend from the Swedish Child Neuropsychiatry Foundation. The remaining authors declare no conflicts of interest.

## Supporting information

Supporting file 1.

## Data Availability

No additional data are available due to Swedish and Norwegian regulations.
