# Supplementary material for: Early‐life adverse events and risk of inflammatory bowel disease: A Scandinavian birth cohort study
Source: J Pediatr Gastroenterol Nutr. 2026 Aug 21;83(5):799–809. doi: 10.1002/jpn3.70537 (PMC13736144; doi:10.1002/jpn3.70537)
Supplement: Supplementary file 1 [file JPN3-83-799-s001.docx]

Supplementary Material

[Supplementary Table 1. Earlier publications on early-life adverse events and IBD risk.	2](#_Toc230167052)

[Supplementary Table 2. Register-based definition of inflammatory bowel disease and its subtypes according to the International Classification of Diseases (ICD)-10 codes.	4](#_Toc230167053)

[Supplementary Table 3. Definitions of measures of early-life adverse events in ABIS and MoBa cohorts, and the harmonization of these variables.	5](#_Toc230167054)

[Supplementary Table 4. Covariates definitions and data sources.	8](#_Toc230167055)

[Supplementary Table 5. Characteristics of individuals with valid data versus those with missing data on early-life adverse events.	9](#_Toc230167056)

[Supplementary Table 6. Characteristics of participants by inflammatory bowel disease, Crohn’s disease, and ulcerative colitis status, based on available cases.	11](#_Toc230167057)

[Supplementary Table 7. Follow-up times, median age at end of follow-up, crude incidence rates, and age- and sex-standardized incidence rates for inflammatory bowel disease, Crohn’s disease, and ulcerative colitis based on available cases in ABIS and MoBa.	13](#_Toc230167058)

[Supplementary Table 8. Associations in secondary analyses between early-life adverse events and inflammatory bowel disease, Crohn’s disease, and ulcerative colitis, using multiple imputation to address missing exposure and covariate data, pooled across cohorts.	14](#_Toc230167059)

[Supplementary Table 9. Associations between early-life adverse events and inflammatory bowel disease, Crohn’s disease, and ulcerative colitis in the ABIS and MoBa cohorts, using available case data pooled across cohorts.	15](#_Toc230167060)

[Supplementary Table 10. Pre-planned sensitivity analyses for associations between early-life adverse events and risk of inflammatory bowel disease, Crohn’s disease, and ulcerative colitis, using multiple imputation of missing exposure and covariate data, pooled across cohorts. Further adjustment was made for breastfeeding duration, diet quality at one year of age, and antibiotic use by one year of age.	16](#_Toc230167061)

[Supplementary Table 11. Associations between early-life adverse events and risk of childhood-onset inflammatory bowel disease (<18 years) in ABIS and MoBa, using multiple imputation of missing exposure and covariate data, pooled across cohorts.	17](#_Toc230167062)

[Supplementary Figure 1. Directed Acyclic Graph (DAG).	18](#_Toc230167063)

[Supplementary Figure 2. Flowchart of study inclusion and exclusion by cohort (ABIS and MoBa), and numbers of individuals included in the main analyses.	19](#_Toc230167064)

[Supplementary Figure 3. Flowchart describing the sample formation for the available-case models.	20](#_Toc230167065)

[References	21](#_Toc230167066)

**Supplementary Table 1.** Earlier publications on early-life adverse events and IBD risk.

Last PubMed search conducted 2025-04-15

| Reference | Year | Country | Study design | Study population | Exposure^a^ | Scale of assessment | Outcome (events) | Findings; point estimate with  95% CI |
| --- | --- | --- | --- | --- | --- | --- | --- | --- |
| Gnat L, et al. Differentiating Childhood Traumas in Inflammatory Bowel Disease. J Can Assoc Gastroenterol. 2023;6(5):172-178. Published 2023 Aug 28. doi:10.1093/jcag/gwad026 | 2023 | Canada | Case-control | Adults.  Mean (SD) age:  Controls: 31.16 (11.52)  IBD patients: 40.48 (12.67) | “Childhood trauma”: prevalence, count, severity, and confiding for six traumas | The Childhood Traumatic Events Scale. | 195 IBD  190 controls | IBD patients reported more severe childhood trauma than controls.  *Controls vs. IBD:*  Total trauma severity: OR 0.89 (0.81–0.97).  Childhood traumatic event by:  Death of a close friend or family OR 0.95 (0.62–1.43);  Extreme illness or injury: OR 0.82 (0.49–1.38) |
| Lee WS, et al. Environmental risk factors for inflammatory bowel disease: A case control study in Southeast Asian children. J Paediatr Child Health. 2022;58(5):782-790. doi:10.1111/jpc.15830 | 2022 | Malaysia | Case-control | Pediatric patients, aged <18 years, and matched controls | “Stressful events” (past or present, examples: generalized anxiety, social anxiety, panic disorders, bereavement, divorce, etc.) | Adapted retrospective questionnaire based on the International Organization for the Study of Inflammatory Bowel Disease opinion on environmental factors of IBD. | 70 children with IBD;  38 CD  32 UC  140 controls | Major stressful events, univariate IBD OR 5.09 (1.84–14.07); CD OR 16.94 (2.00–143.4) [one case in control group]; UC OR 1.22 (0.27–5.46). Multivariate IBD OR 5.55 (1.42–21.77); CD OR 8.37 (0.83–84.59); UC OR not presented. |
| Bednarikova H, Kascakova N, Furstova J, et al. Life Stressors in Patients with Inflammatory Bowel Disease: Comparison with a Population-Based Healthy Control Group in the Czech Republic. Int J Environ Res Public Health. 2021;18(6):3237. Published 2021 Mar 21. doi:10.3390/ijerph18063237 | 2021 | Czech Republic | Case-control | Adolescents & adults. IBD patients; mean age 38 years, range 19–64, and matched controls; mean age 36, range 15–75 | “Life stressors” experienced before 16 years of age (and life stressors reaching traumatic stress, and interpersonal violence), and specified by individual life stress events like before the age of 16 years:  Violence in the family, Physical abuse before the age of 16 years | The Life Stressor Checklist-Revised (LSC-R). | 98 IBD  405 controls | Life stressors before the age of 16 years: IBD OR 2.82 (95%CI 1.39–5.73):  Violence in the family before the age of 16 years: IBD OR 2.51 (1.16–5.44):  Physical abuse before the age of 16 years: IBD OR 4.69 (1.60–13.76)  vs. Healthy controls |
| Strisciuglio C, Giugliano F, Martinelli M, et al. Impact of Environmental and Familial Factors in a Cohort of Pediatric Patients With Inflammatory Bowel Disease. J Pediatr Gastroenterol Nutr. 2017;64(4):569-574. doi:10.1097/MPG.0000000000001297 | 2017 | Italy | Case-control | Younger than 18 years at the time of IBD diagnosis.  Pediatric patients attending the Department for clinical management from 2000 to 2014. | ‘”Stressing events” susceptibility (generalized anxiety, social anxiety, panic disorders, bereavement, divorce). Questions covered the period from birth to the date of diagnosis for cases. | A questionnaire was filled in between January and June 2014. | 102 CD  162 UC  203 controls | A higher percentage of stressing events in the family was found in both groups of cases compared with controls (CD: 46.1% vs controls: 34.5%; χ2: 3.9; P = 0.05; UC: 54.7% vs controls: 34.5%; χ2: 14.9; P < 0.001). |
| Jakobsen C, et al. Environmental factors and risk of developing paediatric inflammatory bowel disease -- a population based study 2007-2009. J Crohns Colitis. 2013;7(1):79-88. doi:10.1016/j.crohns.2012.05.024 | 2013 | Denmark | Case-control | Pediatric patients, aged >3 and <15 years (median age at questionnaire 13 years, range 3–17), and matched controls (median age at questionnaire 10 years, range 3–15) | “Stressful events” (death in family, divorce of parents, moving from one country to another). | An adapted questionnaire developed in 1999 by the International  Organization for the study of Inflammatory Bowel Diseases. | 118 IBD;  59 CD  56 UC  3 IBD-unclassified  477 controls | Risk by Divorced parents:  IBD  Univariate analysis OR 2.4 (1.5-3.7),  Multivariate analysis OR 1.7 (1.0-2.9)  CD  Univariate analysis OR 2.5 (1.4-4.6)  Multivariate analysis NA  UC  Univariate analysis OR 2.2 (1.2-4.1)  Multivariate analysis NA |
| Gilat T, et al. Childhood factors in ulcerative colitis and Crohn's disease. An international cooperative study. Scand J Gastroenterol. 1987;22(8):1009-1024. doi:10.3109/00365528708991950 | 1987 | International  (14 centers in 9 countries) | Case-control | Age at disease onset <20 years, age at time of study <25 years | Major “stressful life events” in childhood (parental death, divorce, severe parental disease or disability, and very poor socioeconomic). | Retrospective questionnaire. | 499 IBD;  302 CD  97 UC | No significant difference between patients and controls in the occurrence of major stressful life events. |

^a^The definitions of early life stress or adversity in previous publications vary and are somewhat overlapping, including stressful/stressing events or life events, adverse events, life stressors, childhood trauma, or childhood adversity, based on varying types of factors on childhood adversity (parental divorce, loss of a family member, violence, abuse, or illnesses, etc.). This study used the term adverse events to refer to experiences of prenatal or early childhood adversities. CD, Crohn's disease; IBD, inflammatory bowel disease; SD, standard deviation; UC, ulcerative colitis.

**Supplementary Table 2.** Register-based definition of inflammatory bowel disease and its subtypes according to the International Classification of Diseases (ICD)-10 codes.

| **Disease** | **ICD-10 (available year 1997 onwards)** | **Outcome definition** |
| --- | --- | --- |
| IBD | CD (K50) / UC (K51) /Indeterminate colitis (K52.3), or a mix of CD/UC the last two years | IBD diagnosis is defined by ≥ 2 inpatient or non-primary outpatient care visits with records of an IBD diagnosis. Validations using this definition have shown a high positive predictive value in Sweden and Norway (1, 2). The first recorded diagnosis defined the onset of disease (3). For patients shifting between CD and UC, the most recently recorded ICD-10 code defined the diagnosis. Patients with a mix of codes for UC, CD, or indeterminate colitis during the last two years of follow-up were classified as (“any, non-specific”) IBD (Indeterminate colitis/IBD-unclassified). |
| CD | K50 |  |
| UC | K51 |  |
| CD, Crohn's disease; IBD, inflammatory bowel disease; ICD-10, International Classification of Diseases; UC, ulcerative colitis. | | |

**Supplementary Table 3.** Definitions of measures of early-life adverse events in ABIS and MoBa cohorts, and the harmonization of these variables.

| Timepoint | MoBa | ABIS | Harmonization |
| --- | --- | --- | --- |
| During pregnancy | **Adverse maternal life events during pregnancy** | | |
|  | Gestational week 30 questionnaire: Q128. Have you experienced any of the following during the last 12 months? (Yes/No)  *Have you been divorced, separated or ended your relationship with your partner?*  *Have you had problems or conflicts with your family, friends or neighbors?*  *Have you been seriously ill or injured?*  *Has anyone close to you been seriously ill or injured?*  *Have you been involved in a serious accident, fire, or robbery?*  *Have you lost someone close to you?*  If Yes, how painful or difficult was it for you?  *1-Not too bad, 2-Painful/difficult, 3-Very painful/difficult* | At birth questionnaire:  Q29: Have you been exposed to, what you consider to be, a difficult life event, during pregnancy (e.g., relative died, divorce)?  *Yes / No*  If Yes, under what month of pregnancy?  *1, 2, 3, 4, 5, 6, 7, 8, and/or 9* | MoBa:  *No, o*r reporting difficulty as  *Not too bad*  *Yes,* if reporting Yes and difficulty as *Painful/difficult* or *Very painful/difficult* (Secondary analyses considered only *Very painful/difficult* events)  ABIS:  *No* (i.e., no adverse event) if reporting No  *Yes,* if reporting Yes or any months of pregnancy (unless answering *No*) |
| By one year  of age | **Adverse family life events by 1–1.5 years** | | |
|  | 6-month questionnaire:  Q91. Have you experienced any of the following situations since the previous questionnaire? (Yes/No)  And/or:  18-month questionnaire:  Q100. Have you experienced any of the following situations in the last year (since the previous questionnaire)? (Yes/No)  *Have you been divorced, separated, or ended your relationship with your partner?*  *Have you had problems or conflicts with family, friends, or neighbors?*  *Have you been seriously ill or injured?*  *Has anyone close to you been seriously ill or injured?*  *Have you been involved in a serious accident, fire or robbery?*  *Have you lost someone close to you?*  *Have you been pressurized into having sexual intercourse?*  If Yes, how painful or difficult was it for you?  *1-Not too bad, 2-Painful/difficult, 3-Very painful/difficult* | 12-month questionnaire:  Q44: Has the child or the family been involved in a serious or dramatic incident (death, divorce, new caregiver, etc)?  *Yes / No / Don't know*  Under what months of life?  *Month 1–2, 3–4, 5–6, 7–8, 9–10, and/or 11–12*  If yes: *Free text answers*  0–12 months diary:  State any important life events/changes (has the child moved, been separated from a close one, had siblings, close one seriously ill or died etc.)?  *Free text answers for up to four events*  *We considered adverse events such as* deaths (within two generations away), divorce/separation, sickness, injuries/accidents, moving, or changes in caregivers. | MoBa:  *No, o*r reporting difficulty as  *Not too bad*    *Yes,* if reporting Yes and difficulty as *Painful/difficult* or *Very painful/difficult -*  *Separately for 6 and 18 months, Yes by one year will consider Yes at either 6 or 18 months.* (Secondary analyses considered only *Very painful/difficult* events)  ABIS:  *No,* if reporting No  *Yes,* if reporting Yes or any months of pregnancy (unless answering *No*), or if reporting adverse events by free text questions. |
| By three years  of age | **Adverse family life events by 2.5–3 years** | | |
|  | 36-month questionnaire:  Q71. Have you experienced during the last 18 months any of the following situations? (Yes/No)  *Have you been divorced, separated, or ended your relationship with your partner?*  *Have you had problems or conflicts with family, friends, or neighbors?*  *Have you been seriously ill or injured?*  *Has anyone close to you been seriously ill or injured?*  *Have you been involved in a serious accident, fire, or robbery?*  *Have you lost someone close to you?*  *Have you been pressurized into having sexual intercourse?*  If Yes, how painful or difficult was it for you?  *1-Not too bad, 2-Painful/difficult, 3-Very painful/difficult* | 2.5–3 years questionnaire:  Q30: Has the child or the family been involved in a serious or dramatic incident (death, divorce, new caregiver etc)?  *Yes / No / Don't know*  Under what month/which months of life?  *Month*  *1–6 , 7–12, 13–18, 19–24, 25–30, and/or 31–36*  2.5–3 years questionnaire:  Q99: Have you been exposed to, what you consider to be, a difficult life event after the child was born?  *Yes / No*  If Yes, what?  *Relative died*  *Serious illness in the family*  *Serious accident in the family*  *Divorce*  *Exposed to violence* | MoBa:  *No, o*r reporting difficulty as  *Not too bad*  *Yes,* if reporting Yes and difficulty as *Painful/difficult* or *Very painful/difficult.* (Secondary analyses considered only *Very painful/difficult* events)  ABIS:  *No,* if reporting No at both questions  *Yes,* at either question, or reporting any month of pregnancy, or reporting a defined event *(unless answering No to the initial questions)* |
| Birth and at three years | **Maternal social support at birth and at three years of age** | | |
|  | Gestational week 30 questionnaire:  Q117. Do you have anyone other than your spouse/boyfriend/partner whom you can seek advice from in a difficult situation?  *No*  *Yes, 1 or 2 people*  *Yes, more than 2 people*  36-month questionnaire:  Q50. Do you have anyone other than your spouse/boyfriend/partner whom you can seek advice from in a difficult situation?  *No*  *Yes, 1 or 2 people*  *Yes, more than 2 people* | At birth questionnaire:  Q30: Do you think that you get the support that you need, for you and your newborn child, from your environment?  *Yes / No*  2.5–3 years questionnaire:  Q100: Do you think that you get the support that you need, for you and your child/children, from your environment?  *On the scale 1–6, where:*  *1 = yes definitely*  *6 = no, not at all* | MoBa:  **Low social support**  *No*  **High social support**  *Yes, 1 or 2 people*  Or *Yes, more than 2 people*  ABIS:  **Low social support**  *Scale 4–6*  **High social support**  *Scale 1–3*  *Separate assessment at birth and at 36 months of age* |
| Birth to 12/18 months of age | **Maternal feelings of depression first year of life** | | |
|  | 6-month questionnaire:  Q92. Have you experienced any of the following feelings during the last week? (*Yes, almost all the time, Yes, now and then, Not very often, No, never)*  *Really reproached yourself when something went wrong*  *Have been anxious or worried for no reason*  *Have been afraid or panicked for no reason*  *Have been so unhappy that you’ve had problems sleeping*  *Felt down or unhappy*  *Have been so unhappy that you’ve cried*  Based on the Edinburgh Postnatal Depression Scale (4), where each feeling is graded by:  *Yes, most of the time = 4 points*  *Yes, some of the time = 3 points*  *Not very often = 2 points*  *No, never = 1 points* | 12-month questionnaire:  Q121. Since I became a parent, I have felt sadder and lower than I expected.  *On the scale 1–6, where:*  1 = I don't agree at all  6 = I agree entirely | MoBa:  Based on the assessment of the Scaled Edinburgh Postnatal Depression Scale (5), where 46% of the total = moderate depression.  **No feelings of depression**  <8 points of the total 18 points  **Yes, feelings of depression**  A score of **≥8 points** of the total 18 points (44%)  ABIS:  **No feelings of depression**  Scale 1–3  **Yes, feelings of depression**  Scale 4–6 |
| ABIS, All Babies in Southeast Sweden; MoBa, The Norwegian Mother, Father, and Child Cohort Study | | | |

# **Supplementary Table 4.** Covariates definitions and data sources.

| Covariate & definition | MoBa | ABIS |
| --- | --- | --- |
| Child’s sex (female or male) | The Medical Birth Registry of Norway | At birth questionnaire |
| Parental IBD (yes or no)  At least one parent with an IBD diagnosis. | The Norwegian Patient Register | At birth questionnaire |
| Parental origin  (Norwegian/Swedish or Other country)  MoBa: Based on parental native language.  ABIS: Based on country of birth.  Considered ”other country” if *at least one parent* reported another native language than Norwegian, or if not being born in Sweden, respectively. | 15-weeks pregnancy questionnaire | At birth questionnaire |
| Maternal age at delivery (categorical)  By six age groups: 15–19, 20–24, 25–29, 30–34, 35–39, 40–44 years (excluding mothers over 45 years) | The Medical Birth Registry of Norway | The Swedish National Medical Birth Registry |
| Maternal education level  (9–11, 12 or ≥13 years)  9–11 years, corresponding to completed compulsory school or ongoing high school.  12 years, corresponding to completed high school or ongoing college or university.  ≥13 years, corresponding to completed college or university, or ongoing higher education. | 15-weeks pregnancy questionnaire | At birth questionnaire |
| Maternal smoking during pregnancy  (yes or no)  Indicate whether any tobacco cigarette use was reported at any point during pregnancy. | 15-weeks & 30-weeks pregnancy questionnaire, and 6-month questionnaire | At birth questionnaire |
| Maternal immune-mediated comorbidities (type 1 diabetes/insulin-treated diabetes, autoimmune thyroid disease, or rheumatoid arthritis) | 15-weeks pregnancy questionnaire | At birth questionnaire |
| ABIS, All Babies in Southeast Sweden; IBD, Inflammatory bowel disease; MoBa, The Norwegian Mother, Father, and Child Cohort Study | | |

# **Supplementary Table 5.** Characteristics of individuals with valid data (≥1 exposure measurement during pregnancy, at age one, or at age three years) versus those excluded due to lack of exposure data at all time points.

|  | **MoBa** | |  | **ABIS** | |
| --- | --- | --- | --- | --- | --- |
| Characteristics | Included (≥1 exposure)  N = 95,220 | Excluded (no exposure data)  N = 6,050 |  | Included (≥1 exposure)  N = 16,073 | Excluded (no exposure data)  N = 150 |
| **Sex** |  |  |  |  |  |
| Male | 48,717 (51%) | 3,153 (52%) |  | 8,334 (52%) | 68 (45%) |
| Female | 46,503 (49%) | 2,897 (48%) |  | 7,739 (48%) | 82 (55%) |
| **Age (years) at the end of follow-up^a^** | |  |  |  |  |
| Mean (SD);  Median (IQR) | 19.4 (2.2);  19.3  (17.7, 21.1) | 19.1 (2.2);  18.8  (17.3, 20.4) |  | 25.2 (1.3);  25.3  (24.8, 25.8) | 25.0 (1.9);  25.2  (24.7, 25.5) |
| **Calendar year of birth** | |  |  |  |  |
| 1997 | – | – |  | 1,655 (10%) | 13 (9%) |
| 1998 | – | – |  | 8,528 (53%) | 74 (49%) |
| 1999 | 30 (<0.1%) | 11 (0.2%) |  | 5,890 (37%) | 63 (42%) |
| 2000 | 1,747 (2%) | 206 (3%) |  | – | – |
| 2001 | 3,680 (4%) | 166 (3%) |  | – | – |
| 2002 | 7,803 (8%) | 304 (5%) |  | – | – |
| 2003 | 11,408 (12%) | 502 (8%) |  | – | – |
| 2004 | 12,252 (13%) | 577 (10%) |  | – | – |
| 2005 | 13,951 (15%) | 956 (16%) |  | – | – |
| 2006 | 15,495 (16%) | 1,058 (17%) |  | – | – |
| 2007 | 14,188 (15%) | 1,081 (18%) |  | – | – |
| 2008 | 11,763 (12%) | 928 (15%) |  | – | – |
| 2009 | 2,903 (3%) | 261 (4%) |  | – | – |
| **IBD diagnosis, including cases diagnosed with IBD after 1 year** | | |  |  |  |
| IBD | 545 (0.6%) | 35 (0.6%) |  | 151 (0.9%) | 1 (0.7%) |
| CD | 227 (0.2%) | 10 (0.2%) |  | 56 (0.3%) | 0 (0%) |
| UC | 184 (0.2%) | 13 (0.2%) |  | 85 (0.5%) | 1 (0.7%) |
| **Any breastfeeding duration** | |  |  |  |  |
| 0–<4 months | 11,492 (13%) | 257 (21%) |  | 1,466 (13%) | 6 (30%) |
| 4–<6 months | 7,640 (9%) | 142 (12%) |  | 1,110 (10%) | 3 (15%) |
| ≥6 months | 67,096 (78%) | 802 (67%) |  | 8,387 (77%) | 11 (55%) |
| Missing | 8,992 | 4,849 |  | 5,110 | 130 |
| **Maternal age^b^** |  |  |  |  |  |
| <25 years | 10,118 (11%) | 953 (16%) |  | 2,517 (16%) | 34 (23%) |
| 25-34 years | 68,420 (72%) | 4,011 (66%) |  | 11,338 (72%) | 102 (69%) |
| 35-44 years | 16,633 (17%) | 1,081 (18%) |  | 1,960 (12%) | 12 (8%) |
| Missing | 49 | 5 |  | 258 | 2 |
| **Maternal education level** |  |  |  |  |  |
| 9–11 years | 7,039 (7%) | 990 (17%) |  | 1,352 (9%) | 6 (17%) |
| 12 years | 27,820 (29%) | 1,915 (32%) |  | 8,794 (55%) | 19 (54%) |
| >13 years | 59,953 (63%) | 3,044 (51%) |  | 5,659 (36%) | 10 (29%) |
| Missing | 408 | 101 |  | 268 | 115 |
| **Parental IBD^c^** |  |  |  |  |  |
| No IBD | 93,004 (98%) | 5,941 (98%) |  | 15,879 (99%) | 149 (99%) |
| IBD | 2,216 (2%) | 109 (2%) |  | 194 (1%) | 1 (0.7%) |
| **Parental origin^d^** |  |  |  |  |  |
| Norwegian/Swedish | 81,985 (90%) | 4,619 (82%) |  | 14,111 (89%) | 31 (84%) |
| Other country | 9,323 (10%) | 1,023 (18%) |  | 1,694 (11%) | 6 (16%) |
| Missing | 3,912 | 408 |  | 268 | 113 |
| **Maternal immune-mediated comorbidities** | |  |  |  |  |
| No | 91,402 (96%) | 5,796 (96%) |  | 15,507 (96%) | 148 (99%) |
| Yes | 3,818 (4%) | 254 (4%) |  | 566 (4%) | 2 (1%) |

^a^Equaled the age at study end (end of 2024 in MoBa; end of 2023 in ABIS) or age at diagnosis for IBD, CD, and UC cases. ^b^Grouped in analyses to six age groups: 15–19, 20–24, 25–29, 30–34, 35–39, 40–44, combined for table presentation. ^c^At least one parent with an IBD diagnosis. ^d^Based on parental native language in MoBa and country of birth in ABIS, and considered ”other country” if at least one parent another native language than Norwegian, or not being born in Sweden, respectively. ABIS, All Babies in Southeast Sweden; CD, Crohn’s disease; IBD, Inflammatory bowel disease; IQR, interquartile range; MoBa, The Norwegian Mother, Father, and Child Cohort Study; UC, ulcerative colitis. See Supplementary Table 4 for covariate definitions and sources. Baseline characteristics were summarised using means with standard deviations (SD) or medians with interquartile ranges (IQR) for continuous variables, and counts with percentages for categorical variables. Percentages ≥1 were rounded to the closest integer.

# **Supplementary Table 6.** Characteristics of participants by inflammatory bowel disease, Crohn’s disease, and ulcerative colitis status, based on available cases.

|  | **MoBa** | | | |  | **ABIS** | | | |
| --- | --- | --- | --- | --- | --- | --- | --- | --- | --- |
| Characteristics | All  N = 95,220 | IBD  n = 546 | CD  n = 227 | UC  n = 185 |  | All  N = 16,073 | IBD  N = 151 | CD N = 56 | UC N = 85 |
| **Sex** | | | | |  |  |  |  |  |
| Male | 48,717 (51%) | 295 (54%) | 130 (57%) | 88 (48%) |  | 8,334 (52%) | 78 (52%) | 29 (52%) | 44 (52%) |
| Female | 46,503 (49%) | 251 (46%) | 97 (43%) | 97 (52%) |  | 7,739 (48%) | 73 (48%) | 27 (48%) | 41 (48%) |
| **Age (year) at the end of follow-up^a^** | | | | |  |  |  |  |  |
| Mean (SD);  Median (IQR) | 19.4 (2.2);  19.3  (17.7, 21.1) | 15.0 (4.1);  15.6  (12.7, 17.8) | 14.8 (3.7);  15.2  (12.5, 17.4) | 16.2 (4.1);  16.9  (14.7, 18.8) |  | 25.2 (1.3);  25.3  (24.8, 25.8) | 18.5 (4.2); 18.7  (15.9, 22.1) | 18.1 (4.0);  18.5  (15.6, 20.8) | 19.0 (4.4);  19.7  (17.0, 22.5) |
| **Calendar year of birth** | |  |  |  |  |  |  |  |  |
| 1997 | – | – | – | – |  | 1,655 (10%) | 13 (9%) | 3 (5%) | 9 (11%) |
| 1998 | – | – | – | – |  | 8,528 (53%) | 88 (58%) | 35 (63%) | 48 (56%) |
| 1999 | 30 (<0.1%) | 0 (0%) | 0 (0%) | 0 (0%) |  | 5,890 (37%) | 50 (33%) | 18 (32%) | 28 (33%) |
| 2000 | 1,747 (2%) | 10 (2%) | 4 (2%) | 3 (2%) |  | – | – | – | – |
| 2001 | 3,680 (4%) | 29 (5%) | 9 (4%) | 14 (8%) |  | – | – | – | – |
| 2002 | 7,803 (8%) | 60 (11%) | 23 (10%) | 22 (12%) |  | – | – | – | – |
| 2003 | 11,408 (12%) | 91 (17%) | 31 (14%) | 39 (21%) |  | – | – | – | – |
| 2004 | 12,252 (13%) | 77 (14%) | 37 (16%) | 24 (13%) |  | – | – | – | – |
| 2005 | 13,951 (15%) | 84 (15%) | 32 (14%) | 32 (17%) |  | – | – | – | – |
| 2006 | 15,495 (16%) | 74 (14%) | 28 (12%) | 23 (12%) |  | – | – | – | – |
| 2007 | 14,188 (15%) | 60 (11%) | 30 (13%) | 12 (7%) |  | – | – | – | – |
| 2008 | 11,763 (12%) | 53 (10%) | 29 (13%) | 12 (7%) |  | – | – | – | – |
| 2009 | 2,903 (3%) | 8 (2%) | 4 (2%) | 4 (2%) |  | – | – | – | – |
| **Adverse maternal life event during pregnancy** | | |  |  |  |  |  |  |  |
| No | 68,896 (75%) | 370 (70%) | 143 (65%) | 134 (74%) |  | 14,312 (91%) | 140 (93%) | 54 (96%) | 79 (94%) |
| Yes | 23,532 (25%) | 159 (30%) | 76 (35%) | 48 (26%) |  | 1,478 (9%) | 10 (7%) | 2 (4%) | 5 (6%) |
| Missing | 2,792 | 17 | 8 | 3 |  | 283 | 1 | 0 | 1 |
| **Adverse family life event by one year of age** | | |  |  |  |  |  |  |  |
| No | 43,183 (58%) | 257 (59%) | 110 (61%) | 84 (56%) |  | 9,122 (85%) | 91 (83%) | 38 (93%) | 49 (79%) |
| Yes | 31,086 (42%) | 180 (41%) | 70 (39%) | 66 (44%) |  | 1,594 (15%) | 19 (17%) | 3 (7%) | 13 (21%) |
| Missing | 20,951 | 109 | 47 | 35 |  | 5,357 | 41 | 15 | 23 |
| **Adverse family life event by three years of age** | | |  |  |  |  |  |  |  |
| No | 26,553 (42%) | 164 (43%) | 71 (44%) | 52 (41%) |  | 4,781 (59%) | 46 (58%) | 17 (68%) | 26 (53%) |
| Yes | 36,328 (58%) | 215 (57%) | 89 (56%) | 76 (59%) |  | 3,383 (41%) | 34 (43%) | 8 (32%) | 23 (47%) |
| Missing | 32,339 | 167 | 67 | 57 |  | 7,909 | 71 | 31 | 36 |
| **Any breastfeeding duration** | | | | |  |  |  |  |  |
| 0–<4 months | 11,492 (13%) | 78 (15%) | 34 (16%) | 25 (15%) |  | 1,466 (13%) | 15 (14%) | 8 (19%) | 6 (11%) |
| 4–<6 months | 7,640 (9%) | 50 (10%) | 24 (11%) | 14 (8.2%) |  | 1,110 (10%) | 14 (13%) | 5 (12%) | 8 (14%) |
| ≥6 months | 67,096 (78%) | 377 (75%) | 155 (73%) | 132 (77%) |  | 8,387 (77%) | 77 (73%) | 29 (69%) | 42 (75%) |
| Missing | 8,992 | 41 | 14 | 14 |  | 5,110 | 45 | 14 | 29 |
| **Maternal age^b^** | | | | |  |  |  |  |  |
| <25 years | 10,118 (11%) | 56 (10%) | 18 (7.9%) | 21 (11%) |  | 2,517 (16%) | 19 (13%) | 8 (15%) | 9 (11%) |
| 25-34 years | 68,420 (72%) | 390 (71%) | 167 (74%) | 130 (70%) |  | 11,338 (72%) | 111 (75%) | 37 (69%) | 66 (79%) |
| 35-44 years | 16,633 (17%) | 100 (18%) | 42 (19%) | 34 (18%) |  | 1,960 (12%) | 18 (12%) | 9 (17%) | 9 (11%) |
| Missing | 49 | 0 | 0 | 0 |  | 258 | 3 | 2 | 1 |
| **Maternal education level** | | | | |  |  |  |  |  |
| 9–11 years | 7,039 (7%) | 50 (9.2%) | 19 (8.4%) | 15 (8.2%) |  | 1,352 (9%) | 16 (11%) | 6 (11%) | 9 (11%) |
| 12 years | 27,820 (29%) | 158 (29%) | 57 (25%) | 61 (33%) |  | 8,794 (55%) | 87 (58%) | 33 (59%) | 50 (59%) |
| >13 years | 59,953 (63%) | 334 (62%) | 150 (66%) | 107 (58%) |  | 5,659 (36%) | 48 (32%) | 17 (30%) | 26 (31%) |
| Missing | 408 | 4 | 1 | 2 |  | 268 | 0 | 0 | 0 |
| **Parental IBD^c^** |  |  |  |  |  |  |  |  |  |
| No IBD | 93,004 (98%) | 531 (97%) | 220 (97%) | 183 (99%) |  | 15,879 (99%) | 142 (94%) | 52 (93%) | 81 (95%) |
| IBD | 2,216 (2%) | 15 (3%) | 7 (3%) | 2 (1%) |  | 194 (1%) | 9 (6%) | 4 (7%) | 4 (5%) |
| **Parental origin^d^** | | | | |  |  |  |  |  |
| Norwegian/Swedish | 81,985 (90%) | 468 (89%) | 200 (91%) | 157 (90%) |  | 14,111 (89%) | 132 (88%) | 52 (93%) | 71 (85%) |
| Other country | 9,323 (10%) | 55 (11%) | 20 (9%) | 17 (10%) |  | 1,694 (11%) | 18 (12%) | 4 (7%) | 13 (15%) |
| Missing | 3,912 | 23 | 7 | 11 |  | 268 | 1 | 0 | 1 |
| **Maternal immune-mediated comorbidities** | | |  |  |  |  |  |  |  |
| No | 91,402 (96%) | 526 (96%) | 219 (96%) | 176 (95%) |  | 15,507 (96%) | 146 (97%) | 55 (98%) | 81 (95%) |
| Yes | 3,818 (4%) | 20 (4%) | 8 (4%) | 9 (5%) |  | 566 (4%) | 5 (3%) | 1 (2%) | 4 (5%) |

^a^Equaled the age at study end (end of 2024 in MoBa; end of 2023 in ABIS) or age at diagnosis for IBD, CD, and UC cases. ^b^Grouped in analyses to six age groups: 15–19, 20–24, 25–29, 30–34, 35–39, 40–44, combined for table presentation. ^c^At least one parent with an IBD diagnosis. ^d^Based on parental native language in MoBa and country of birth in ABIS, and considered ”other country” if at least one parent reported another native language than Norwegian, or not being born in Sweden, respectively. ABIS, All Babies in Southeast Sweden; CD, Crohn’s disease; IBD, Inflammatory bowel disease; IQR, interquartile range; MoBa, The Norwegian Mother, Father, and Child Cohort Study; UC, ulcerative colitis. See Supplementary Table 4 for covariate definitions and sources. Baseline characteristics were summarised using means with standard deviations (SD) or medians with interquartile ranges (IQR) for continuous variables, and counts with percentages for categorical variables. Percentages ≥1 were rounded to the closest integer.

# **Supplementary Table 7.** Follow-up times, median age at end of follow-up, crude incidence rates, and age- and sex-standardized incidence rates for inflammatory bowel disease, Crohn’s disease, and ulcerative colitis based on available cases in ABIS and MoBa.

|  | n Events  (% of total population) | Follow-up time  (person-years) | Median age at end of follow-up (IQR) | Incidence per  100'000 PY  (95% CI) | Age- and sex-  standardized incidence per  100'000 PY (95% CI)^a^ |
| --- | --- | --- | --- | --- | --- |
| **MoBa** |  |  | | |  |
| *IBD* | 546 (0.6) | 1 850 700 | 15.6 (12.7, 17.8) | 29.5 (27.1–32.1) | – |
| *CD* | 227 (0.2) | 1 852 315 | 15.2 (12.5, 17.4) | 12.3 (10.7–14.0) | – |
| *UC* | 185 (0.2) | 1 852 679 | 16.9 (14.7, 18.8) | 10.0 (8.6–11.5) | – |
| **ABIS** |  |  | | |  |
| *IBD* | 151 (0.9) | 404 942 | 18.7 (15.9, 22.1) | 37.3 (31.6–43.7) | 28.1 (19.3–40.9) |
| *CD* | 56 (0.3) | 405 574 | 18.5 (15.6, 20.8) | 13.8 (10.4–17.9) | 11.8 (7.0–19.7) |
| *UC* | 85 (0.5) | 405 439 | 19.7 (17.0, 22.5) | 21.0 (16.8–25.9) | 13.7 (9.4–19.9) |

^a^Incidence rates were calculated as the number of incident IBD cases divided by total person-years of follow-up. Age- and sex-standardized incidence rates were computed using direct standardization, weighting stratum-specific incidence rates according to the age–sex distribution of the MoBa cohort. ABIS, All Babies in Southeast Sweden; IQR, interquartile range; MoBa, The Norwegian Mother, Father, and Child Cohort Study

# **Supplementary Table 8.** Associations in secondary analyses between early-life adverse events and inflammatory bowel disease, Crohn’s disease, and ulcerative colitis, using multiple imputation to address missing exposure and covariate data, pooled across cohorts.

|  | **IBD** | | | **CD** | | | | **UC** | | | | |
| --- | --- | --- | --- | --- | --- | --- | --- | --- | --- | --- | --- | --- |
|  | N (%; n events) | Crude HR  (95% CI) | Adjusted HR  (95% CI)^a^ | N (%; n events) | | Crude HR  (95% CI) | Adjusted HR  (95% CI)^a^ | N (%; n events) | | Crude HR  (95% CI) | Adjusted HR  (95% CI)^a^ |  |
| **Adverse maternal life  event during pregnancy** | | |  |  | |  |  |  | |  |  |  |
| **MoBa^b^** | |  |  |  | |  |  |  | |  |  |  |
| *No* | 85 934 (91; 490) | (reference) | (reference) | 85 934 (91; 205) | | (reference) | (reference) | 85 934 (91; 169) | | (reference) | (reference) |  |
| *Yes* | 8 966 (9; 54) | 1.04 (0.78–1.39) | 1.04 (0.78–1.39) | 8 966 (9; 20) | | 0.92 (0.58–1.47) | 0.95 (0.59–1.52) | 8 966 (9; 15) | | 0.85 (0.50–1.44) | 0.83 (0.49–1.41) |  |
| **Pooled estimates** | |  |  |  | |  |  |  | |  |  |  |
| *No* | 100 503 (91; 630) | (reference) | (reference) | 100 503 (91; 259) | | (reference) | (reference) | 100 503 (91; 250) | | (reference) | (reference) |  |
| *Yes* | 10 470 (9; 64) | 0.95 (0.67–1.33) | 0.94 (0.66–1.33) | 10 470 (9; 22^b^) | | 0.74 (0.34–1.61) | 0.76 (0.34–1.67) | 10 470 (9; 21) | | 0.78 (0.50–1.24) | 0.76 (0.48–1.21) |  |
| **Adverse family life event  by one year** | | |  |  |  |  |  |  |  |  |  |  |
| **MoBa^c^** | |  |  |  |  |  |  |  |  |  |  |  |
| *No* | 77 636 (82; 438) | (reference) | (reference) | 77 636 (82; 188) | | (reference) | (reference) | 77 634 (82; 142) | | (reference) | (reference) |  |
| *Yes* | 17 263 (18; 105) | 1.08 (0.85–1.36) | 1.07 (0.84–1.35) | 17 263 (18; 37) | | 0.89 (0.61–1.31) | 0.92 (0.63–1.36) | 17 265 (18; 42) | | 1.33 (0.90–1.94) | 1.30 (0.88–1.92) |  |
| **Pooled estimates** | |  |  |  | |  |  |  | |  |  |  |
| *No* | 91 128 (82; 564) | (reference) | (reference) | 91 127 (82; 239) | | (reference) | (reference) | 91 127 (82; 210) | | (reference) | (reference) |  |
| *Yes* | 19 839 (18; 130) | 1.07 (0.87–1.32) | 1.07 (0.86–1.32) | 19 840 (18; 42) | | 0.83 (0.56–1.24) | 0.87 (0.59–1.26) | 19 840 (18; 59) | | 1.31 (0.95–1.82) | 1.29 (0.92–1.79) |  |
| **Adverse family life event  by three years** | | |  |  | |  |  |  |  |  |  |  |
| **MoBa^b^** | |  |  |  | |  |  |  |  |  |  |  |
| *No* | 72 968 (77; 414) | (reference) | (reference) | 72 968 (77; 174) | | (reference) | (reference) | 72 967 (77; 138) | | (reference) | (reference) |  |
| *Yes* | 21 926 (23; 124) | 0.99 (0.79–1.24) | 0.99 (0.79–1.24) | 21 926 (23; 50) | | 0.96 (0.67–1.38) | 1.00 (0.69–1.43) | 21 927 (23; 45) | | 1.09 (0.75–1.59) | 1.07 (0.73–1.56) |  |
| **Pooled estimates** | |  |  |  | |  |  |  | |  |  |  |
| *No* | 82 974 (75; 508) | (reference) | (reference) | 82 973 (75; 214) | | (reference) | (reference) | 82 973 (75; 186) | | (reference) | (reference) |  |
| *Yes* | 27 983 (25; 180) | 0.99 (0.82–1.20) | 0.98 (0.81–1.19) | 27 984 (25; 66) | | 0.88 (0.63–1.23) | 0.88 (0.59–1.31) | 27 984 (25; 81) | | 1.14 (0.85–1.54) | 1.12 (0.83–1.51) |  |

^a^Adjusted for child’s sex, parental IBD and origin, maternal age at delivery, educational level, smoking during pregnancy, and comorbidities. ^b^Two events in ABIS, crude model. ^c^Secondary analyses included the more stringent definition of adverse life events by only including events reported as “Very painful/difficult” from the MoBa cohort. No changes were made to the definition of adverse life events in the All Babies in Southeast Sweden (ABIS) cohort. CI, confidence interval; HR, hazard ratio; MoBa, The Norwegian Mother, Father, and Child Cohort Study

# **Supplementary Table 9.** Associations between early-life adverse events and inflammatory bowel disease, Crohn’s disease, and ulcerative colitis in the ABIS and MoBa cohorts, using available case data pooled across cohorts.

|  | **IBD** | | | **CD** | | | | **UC** | | | | |
| --- | --- | --- | --- | --- | --- | --- | --- | --- | --- | --- | --- | --- |
|  | N (n events) | Crude HR  (95% CI) | Adjusted HR  (95% CI)^a^ | N (n events) | | Crude HR  (95% CI) | Adjusted HR  (95% CI)^a^ | N (n events) | | Crude HR  (95% CI) | Adjusted HR  (95% CI)^a^ |  |
| **Adverse maternal life event during pregnancy** | |  |  |  | |  |  |  | |  |  |  |
| No | 83 208 (510) | (reference) | (reference) | 83 208 (197) | | (reference) | (reference) | 83 208 (213) | | (reference) | (reference) |  |
| Yes | 25 010 (169) | 1.01 (0.58–1.76) | 1.02 (0.60–1.74) | 25 010 (78^b^) | | 0.88 (0.22–3.55) | 0.92 (0.24–3.52) | 25 010 (53) | | 0.95 (0.65–1.39) | 0.94 (0.65–1.36) |  |
| **Adverse family life event  by one year** | |  |  |  |  |  |  |  |  |  |  |  |
| No | 52 305 (348) | (reference) | (reference) | 52 305 (148) | | (reference) | (reference) | 52 305 (133) | | (reference) | (reference) |  |
| Yes | 32 680 (199) | 0.99 (0.83–1.19) | 0.97 (0.81–1.17) | 32 680 (73^c^) | | 0.81 (0.53–1.24) | 0.87 (0.66–1.17) | 32 680 (79) | | 1.17 (0.88–1.55) | 1.16 (0.81–1.68) |  |
| **Adverse family life event  by three years** | |  |  |  | |  |  |  |  |  |  |  |
| No | 31 333 (209) | (reference) | (reference) | 31 333 (88) | | (reference) | (reference) | 31 333 (78) | | (reference) | (reference) |  |
| Yes | 39 706 (245) | 0.94 (0.78–1.14) | 0.94 (0.78–1.14) | 39 706 (96) | | 0.86 (0.64–1.15) | 0.90 (0.67–1.21) | 39 706 (98) | | 1.08 (0.80–1.45) | 1.08 (0.79–1.47) |  |

^a^Adjusted for child’s sex, parental IBD and origin, maternal age at delivery, educational level, smoking during pregnancy, and comorbidities. ^b^Two events in ABIS, crude model. ^c^Three events in ABIS, crude model. ABIS, All Babies in Southeast Sweden; CI, confidence interval; HR, hazard ratio; MoBa, The Norwegian Mother, Father, and Child Cohort Study

# **Supplementary Table 10.** Pre-planned sensitivity analyses for associations between early-life adverse events and risk of inflammatory bowel disease, Crohn’s disease, and ulcerative colitis, using multiple imputation of missing exposure and covariate data, pooled across cohorts. Further adjustment was made for breastfeeding duration, diet quality at one year of age, and antibiotic use by one year of age.

|  | **IBD** | | | **CD** | | | | **UC** | | | | |
| --- | --- | --- | --- | --- | --- | --- | --- | --- | --- | --- | --- | --- |
|  | N (n events)^a^ | Crude HR  (95% CI) | Adjusted HR (95% CI)^b^ | N (n events)^a^ | | Crude HR  (95% CI) | Adjusted HR  (95% CI)^b^ | N (n events)^a^ | | Crude HR  (95% CI) | Adjusted HR (95% CI)^b^ |  |
| **Adverse maternal life events during pregnancy** | |  |  |  | |  |  |  | |  |  |  |
| No | 85 454 (522) | (reference) | (reference) | 85 454 (202) | | (reference) | (reference) | 85 455 (216) | | (reference) | (reference) |  |
| Yes | 25 839 (175) | 1.01 (0.58–1.75) | 0.99 (0.55–1.78) | 25 839 (81^c^) | | 0.89 (0.22–3.57) | 0.89 (0.22–3.61) | 25 839 (54) | | 0.96 (0.67–1.37) | 0.92 (0.58–1.44) |  |
| **Adverse family life events  by one year** | |  |  |  |  |  |  |  |  |  |  |  |
| No | 68 335 (445) | (reference) | (reference) | 68 334 (188) | | (reference) | (reference) | 68 337 (172) | | (reference) | (reference) |  |
| Yes | 42 952 (251) | 0.97 (0.81–1.15) | 0.95 (0.80–1.13) | 42 953 (95) | | 0.84 (0.58–1.21) | 0.79 (0.48–1.29) | 42 951 (97) | | 1.08 (0.81–1.44) | 1.09 (0.82–1.45) |  |
| **Adverse family life events  by three years** | |  |  |  | |  |  |  |  |  |  |  |
| No | 56 974 (361) | (reference) | (reference) | 56 972 (152) | | (reference) | (reference) | 56 973 (136) | | (reference) | (reference) |  |
| Yes | 54 303 (329) | 0.99 (0.84–1.17) | 0.98 (0.83–1.15) | 54 305 (130) | | 0.90 (0.62–1.31) | 0.94 (0.65–1.35) | 54 304 (131) | | 1.11 (0.84–1.48) | 1.08 (0.82–1.42) |  |

^a^Mean value based on 20 imputations. ^b^Adjusted for child’s sex, parental IBD and origin, maternal age at delivery, educational level, smoking during pregnancy, comorbidities, breastfeeding duration, diet quality at one year age, and antibiotic use by one year age. ^c^Two events in ABIS, crude model. ABIS, All Babies in Southeast Sweden; CI, confidence interval; HR, hazard ratio; MoBa, The Norwegian Mother, Father, and Child Cohort Study

# **Supplementary Table 11.** Associations between early-life adverse events and risk of childhood-onset inflammatory bowel disease (<18 years) in ABIS and MoBa, using multiple imputation of missing exposure and covariate data, pooled across cohorts.

|  | **Pooled estimates** | | | |
| --- | --- | --- | --- | --- |
|  | N^a^ | n  events^a^ | Crude HR  (95% CI) | Adjusted HR  (95% CI)^b^ |
| **Adverse maternal life  events during pregnancy** | |  |  |  |
| No | 85 456 | 344 | (reference) | (reference) |
| Yes | 25 837 | 139 | 1.00 (0.40–2.49) | 0.99 (0.39–2.52) |
| **Adverse family life events by one year** |  |  |  |  |
| No | 68 352 | 296 | (reference) | (reference) |
| Yes | 42 935 | 186 | 0.88 (0.52–1.48) | 0.88 (0.53–1.46) |
| **Adverse family life events by three years** |  |  |  |  |
| No | 56 970 | 237 | (reference) | (reference) |
| Yes | 54 307 | 239 | 1.05 (0.85–1.29) | 1.04 (0.85–1.28) |

^a^Mean value based on 20 imputations. ^b^Adjusted for child’s sex, parental IBD and origin, maternal age at delivery, educational level, smoking during pregnancy, and comorbidities. CI, confidence interval; HR, hazard ratio

# **Supplementary Figure 1**. Directed Acyclic Graph (DAG).

Directed acyclic graph (DAG) describing the covariates selected for analyses on early-life adverse events and risk of inflammatory bowel disease (IBD). The DAG was created at www.dagitty.net.

# **Supplementary Figure 2.** Flowchart of study inclusion and exclusion by cohort (ABIS and MoBa), and numbers of individuals included in the main analyses.

^a^Including IBD-unclassified. The number of participants varies across exposures due to loss to follow-up over time. ABIS, All Babies in Southeast Sweden; MoBa, The Norwegian Mother, Father, and Child Cohort Study.

# **Supplementary Figure 3**. Flowchart illustrating sample selection for the available-case analyses.

^a^Including IBD-unclassified. The number of participants varies across exposures due to loss to follow-up over time. ABIS, All Babies in Southeast Sweden; MoBa, The Norwegian Mother, Father, and Child Cohort Study.

# **References**

1	Larsen JH, Andersen S, Perminow G, et al. Higher incidence of paediatric inflammatory bowel disease by increasing latitude in Norway, but stable incidence by age. Acta Paediatr 2024;113(7):1720-27.

2	Mouratidou N, Malmborg P, Järås J, et al. Identification of Childhood-Onset Inflammatory Bowel Disease in Swedish Healthcare Registers: A Validation Study. Clin Epidemiol 2022;14(591-600.

3	Shrestha S, Olén O, Eriksson C, et al. The use of ICD codes to identify IBD subtypes and phenotypes of the Montreal classification in the Swedish National Patient Register. Scand J Gastroenterol 2020;55(4):430-35.

4	Cox JL, Holden JM, Sagovsky R Detection of postnatal depression. Development of the 10-item Edinburgh Postnatal Depression Scale. Br J Psychiatry 1987;150(782-6.

5	Eberhard-Gran M, Eskild A, Samuelsen SO, Tambs K A short matrix-version of the Edinburgh Depression Scale. Acta Psychiatr Scand 2007;116(3):195-200.
